# Effect of Crystal Orientation on Self-Assembly Nanocones Formed on Tungsten Surface Induced by Helium Ion Irradiation and Annealing

**DOI:** 10.3390/nano6110210

**Published:** 2016-11-12

**Authors:** Shilin Huang, Guang Ran, Penghui Lei, Shenghua Wu, Nanjun Chen, Ning Li

**Affiliations:** 1 College of Energy, Xiamen University, Xiamen 361102, China; hslxmu@foxmail.com (S.H.); p.h.lei@foxmail.com (P.L.); njchen@umich.edu (N.C.); ningli@xmu.edu.cn (N.L.); 2 School of Materials Science and Engineering, Xi’an Jiaotong University, Xi’an 710049, China; lyyzwsh@163.com

**Keywords:** nanocone, self-assembly, tungsten, irradiation, crystal orientation

## Abstract

The self-assembly nanocone structures on the surface of polycrystalline tungsten were created by He^+^ ion irradiation and then annealing, and the resulting topography and morphology were characterized using atomic force microscopy and scanning electron microscopy. The cross-sectional samples of the self-assembly nanocones were prepared using an in situ–focused ion beam and then observed using transmission electron microscopy. The self-assembly nanocones were induced by the combined effect of He^+^ ion irradiation, the annealing process and the chromium impurity. The distribution characteristics, density and morphology of the nanocones exhibited a distinct difference relating to the crystal orientations. The highest density of the nanocones was observed on the grain surface with a (1 1 1) orientation, with the opposite for that with a (0 0 1) orientation and a medium value on the (1 0 1)-oriented grain. The size of the self-assembly nanocones increased with increasing the annealing time which met a power-law relationship. Irradiation-induced defects acted as the nucleation locations of the protrusions which attracted the migration of the tiny amount of chromium atoms. Under the action of temperature, the protrusions finally evolved into the nanocones.

## 1. Introduction

Ion irradiation can induce some characteristic microstructures with high periodicity such as nanofibers, nanopyramids, nanodots and nanoripples self-assembled on sample surfaces [1,2,3,4]. These periodic self-assembly microstructures with a nano size have excellent photon, phonon and electron properties, which are usually considered as potential applications in nanoscale sensors, phonon devices, electron devices and photonic devices by utilizing their unique physical and chemical properties [5,6,7,8]. The morphologies and topographies of periodic self-assembly microstructures are significantly influenced by the nature of the substrate material, the parameters of ion irradiation such as ion species, fluence and irradiation temperature, and the subsequent annealing process which can be precisely controlled [9,10,11]. Therefore, ion irradiation technology can be used to prepare a periodic self-assembly nanostructure with accurate shape and size on a solid surface for a specific application. It is well known that tungsten is widely used in electron devices, X-ray devices and field emission devices because of its high thermal conductivity, high luminous efficiency, low evaporation rate, low sputtering yield and high melting temperature [12,13], making tungsten a leading candidate suitable for high temperature, high radiation, and extreme environmental conditions.

The self-assembly surface nanostructures induced by ion irradiation have been widely investigated in experiments and theories in the past decades in order to reveal the fundamental physical mechanism. The investigations conducted so far about the formation mechanism of these self-assembly surface nanostructures can be mainly concluded as: (i) the results of dynamic competition among different surface processes during ion beam sputtering, some of these processes induce surface roughening (e.g., sputtering atoms off the surface) while others induce smoothing (e.g., diffusion of atoms); (ii) when ions impinge on a surface, a series of collisions result in the creation of numerous defects both in the bulk (vacancies and interstitials) and on the surface (adatoms and surface vacancies). The formation, growth and coarsening of defects in the materials are important factors controlling the surface morphology, especially during subsequent annealing treatment. The theories about ion beam sputtering in the mechanism (i) have been widely investigated. A lot of models are proposed based on the original BH model (proposed by Bradley and Harper suggesting that local sputtering yield depends on the surface curvature) [14] and ES barrier (the Ehrlich-Schwoebel barriers can induce mass flow and act as a driving force for surface pattern formation) [15,16]. The different sputtering temperature may give a transition between erosive and diffusive regimes. For the mechanism (ii), in fact, some studies have been devoted to the role of annealing in the action of defects and morphology evolution during post-irradiation annealing. Yajima [17] investigated the deformation of the fiber-form nanostructures and the dynamic behavior of helium bubbles in a fuzz tungsten, revealing the coalescence of the fine structure and the annihilation of helium bubbles at around 1073–1173 K. Hino [18] studied helium-ion etching for polycrystalline copper and found that the increase of the surface roughness was attributed to the formation of blisters during ion irradiation. These blisters would disappear and then the sample surface went back to being smooth after the post-irradiation annealing. Kajita [19] reported the formation of nanostructures on a tungsten surface induced by helium plasma irradiation and the evolution of nanostructures during post-irradiation annealing. Concerned with the possible application of specific nanostructures in a high-temperature environment, the investigation of nanostructure evolution at the post-irradiation annealing conditions is necessary.

While studying the irradiation-induced self-assembly nanostructures, the crystal orientation has been found to be an important factor that should be considered. For example, Contantini [20] compared the periodic structures induced by the normal-incidence sputtering on Ag (1 1 0) and Ag (0 0 1) surfaces. The results showed that a periodic pattern of ripples was produced on the Ag (1 1 0) surface, but a periodic pattern of square islands grew on the Ag (0 0 1) surface. Parish [21] reported various surface morphologies such as smooth, wavy, pyramidal and terraced waves formed on a polycrystalline tungsten surface dependent on crystal orientation after 80 eV helium ion bombardment at 1130 °C. Meanwhile, Liu [22] used a phase-field model to investigate the effect of the surface-energy anisotropy of polycrystalline material on the void evolution during ion irradiation. In addition, the influence of the surface chemical composition and impurity element should also be considered, which played an important role during the formation of surface nanostructures induced by ion irradiation [23,24,25,26]. In those reports, the impure atoms are mainly achieved via assisted metal atom co-deposition during the ion-beam irradiation, and they can act as surfactant atoms, strongly modifying the sputtering yields of the substrate and influencing the diffusion and migration of various irradiation-induced defects. Nevertheless, all of these impurities are always introduced from an ion source or sample surroundings; the situation where the tiny amount of impure atoms dissolved in the target material as solid-solution atoms during the process of material preparation is not considered. Therefore, the natural impurity in the target materials should be considered in investigating the mechanism of self-assembly nanostructures induced by ion irradiation and then annealing, which is also a quite challenging and valuable work.

In the present work, helium ion irradiation was used to induce self-assembly nanostructures on a tungsten surface. The effect of the crystal orientation, the annealing process and the tiny amount of chromium impurity on the formation of self-assembly nanostructures were investigated. The corresponding mechanism was also analyzed and discussed.

## 2. Experiments

The polycrystalline tungsten (purity > 99.8 wt %, provided by Xiamen Tungsten Corporation of China, Xiamen, Fujian, China) fabricated by powder metallurgy was used as the original material in the present work. The chemical composition of this polycrystalline tungsten was also tested using inductively coupled plasma mass spectrometry (ICP-MS) according to QB-GP-29-2008 test standard of China at the laboratory of National Center of Analysis Testing for Non-ferrous Metals & Electronic Materials in China (Beijing, China). The test results showed that the content of chromium impurity was less than 0.2 wt %. The samples with 10 mm × 10 mm × 3 mm were first cut from the as-received round rod by a precision diamond knife-cutting machine (Isomet Low Speed Diamond Saw, BUEHLER, Lake Bluff, IN, USA) and then grinded by SiC sandpaper (Beijing Zhongxing Bairui Technology Co. Ltd., Beijing, China) from 320 to 2500 grid, mechanically polished by 3–0.1 µm diamond paste. After that, the samples were finally electrochemical polished by a 2% NaOH aqueous solution in a BUEHLER ELECTROMET^®^4 instrument (BUEHLER, Lake Bluff, IN, USA) to remove surface damage for electron back-scattered diffraction (EBSD) test and then ion irradiation experiment. Before He^+^ ion irradiation, orientation imaging microscopy (OIM) based on EBSD of the polished tungsten was performed using a TESCAN MIRA3 field emission scanning electron microscope (FESEM) (TESCAN, Brno, Czech Republic). The crystal orientations of tungsten grains were then obtained by processing these data.

**Figure 1:**
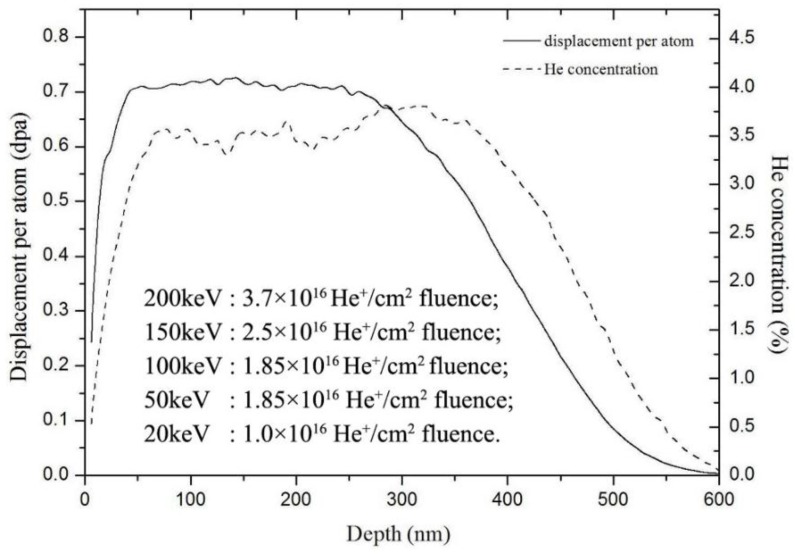
Depth profile of displacement damage and helium concentration in polycrystalline tungsten irradiated by He^+^ ions from 200–20 keV energy with corresponding ion fluence calculated by SRIM 2008 software with quick mode. The atom displacement threshold energy (*E_d_*) of tungsten is 90 eV.

He^+^ ion irradiation was done at normal incidence to the sample surface and was carried out at room temperature using a 400 kV ion implanter (National Electrostatics Corp., Middleton, WI, USA) at our research group. The ion flux was kept at about 2.0 × 10^12^ ions/cm^2^·s to prevent excessive beam heating. He^+^ ion irradiation with a series of energy and corresponding ion fluence was designed to obtain a relatively homogenous displacement damage from sample surface to a desired depth, which was shown in Figure 1. The schedule of the helium-ion irradiation was shown as:

- 200 keV: 3.7 × 10^16^ He^+^/cm^2^ fluence;
- 150 keV: 2.5 × 10^16^ He^+^/cm^2^ fluence;
- 100 keV: 1.85 × 10^16^ He^+^/cm^2^ fluence;
- 50 keV: 1.85 × 10^16^ He^+^/cm^2^ fluence;
- 20 keV: 1.0 × 10^16^ He^+^/cm^2^ fluence.

The helium ions with 200 keV were first used to irradiate the samples. And then ion energy was degraded until 20 keV. According to the simulation results of SRIM 2008 software (SRIM.EXE, (C) 1984–2013, James F. Ziegler, Annapolis, MD, USA) with quick mode, the helium-ion irradiation with a series of energy and fluence caused a 3.5% platform concentration of helium ions in the tungsten sample at the range of 70–400 nm depth. The platform displacement damage was about 0.7 dpa at the range of 50–300 nm depth as shown in Figure 1. The atom displacement threshold energy (*E_d_*) of tungsten was designed to be 90 eV.

After He^+^ ion irradiation with a series of energy and corresponding ion fluence as shown in Figure 1, the irradiated samples were annealed at 900 °C for 0.5 h, 1 h and 3 h. In order to avoid oxidation and contamination during the annealing process, the experiments were carried out at a vacuum furnace with pressure of <10^−4^ mbar. The surface roughness of the annealed samples was measured in air at room temperature using atomic force microscopy (AFM) with a BRUKER Dimension Icon AFM (BRUKER Corporation, Karlsruhe, Germany), operating in a tapping mode using phosphorus doped Si cantilevers. Three-dimensional (3D) topography images were obtained by processing the original AFM data using NanoScope Analysis software (BRUKER Corporation, Karlsruhe, Germany). In order to analyze the characteristic microstructure on the sample surface induced by ion irradiation and annealing, the cross-sectional TEM (transmission electron microscopy) samples were prepared at the location of the characteristic microstructure using in-situ focused ion beam (FIB) ‘lift-out’ technology in a FESEM/FIB dual-beam system. Pt and Au with about 1.0 µm thickness used as a protective layer were first deposited on the sample surfaces during the preparation of TEM sample. The microstructure was analyzed by transmission electron microscopy in a JEM 2100 instrument (JEOL, Tokyo, Japan).

## 3. Results and Discussion

**Figure 2:**
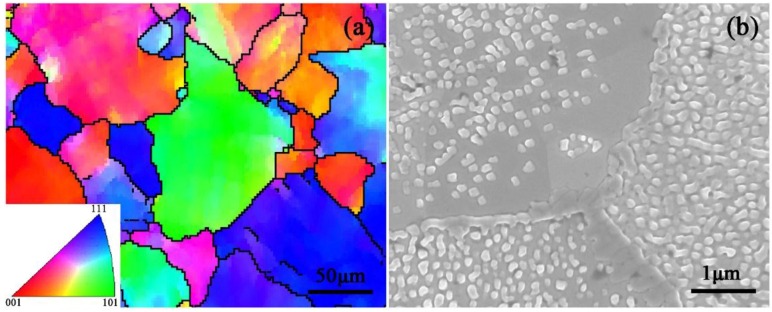
(**a**) Crystal orientation image of the as-polished tungsten obtained by processing electron back-scattered diffraction (EBSD) data; (**b**) Scanning electron microscopy (SEM) image showing the morphology of the tungsten surface after He^+^ ion irradiation with a series of energy degraded from 200 keV to 20 keV and then annealing for 3 h at 900 °C.

The crystal orientation image of the as-polished tungsten is obtained by processing EBSD data as shown in Figure 2a. Various colors in the image represent tungsten grains with different crystal orientations. In addition, crystal boundaries can be obviously observed. The crystal size is in the range of tens of microns to hundreds of micrometers. However, we only focused on the microstructural evolution of tungsten grains with a crystal size over 100 microns during the ion irradiation and annealing process. From the original test data, we can find tungsten grains with planes parallel to the sample surface in the interested area such as (0 0 1)-, (1 0 1)- and (1 1 1)-oriented grains.

The typical surface morphology of the irradiated sample annealed for 3 h at 900 °C is shown in Figure 2b. It can be seen that a lot of characteristic microstructures with a white dot shape (named a nanocone) are distributed on the sample surface. In addition, some characteristic structures are also shown as short rod-like structures, which is due to the growth and combination of the nanocones during the annealing process. Because the observation direction is perpendicular to the sample surface, the microstructures with those characteristic morphologies are displayed on the SEM image. Crystal boundaries can be clearly observed which distinguish three grains with different crystal orientations. Interestingly, the density and distribution characteristics of the nanocones are obviously different in the tungsten grains with different crystal orientations, which shows that the crystal orientation influences the formation and growth of the nanocones. In addition, the distribution characteristics of the nanocones in an individual grain is relatively uniform. However, actually, in some grains, there are few nanocones distributing near the grain boundaries, but many far away from the grain boundaries, as shown in the upper left corner of Figure 2b.

After mechanically and then electrochemically being polished, the whole sample surface has the same roughness of about 3 nm as shown in the AFM test data. The surface morphology of the as-polished polycrystalline tungsten is similar, although the crystal orientation is different. Therefore, the self-assembly nanocones are induced by the helium-ion irradiation and subsequent annealing. The morphology and characteristics of the nanocones are mainly influenced by the crystal orientation under the same experimental conditions.

**Figure 3:**
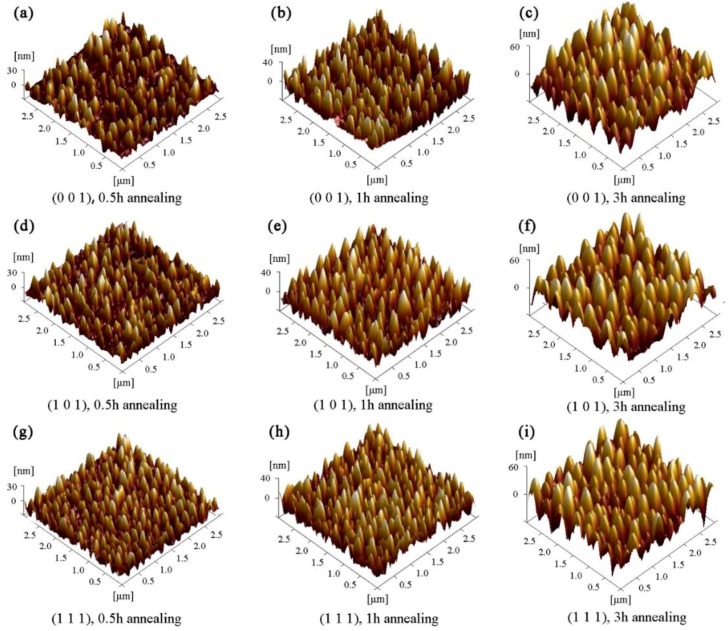
Atomic force microscopy (AFM) 3D images of the irradiated tungsten surface with (0 0 1) orientation after (**a**) 0.5 h, (**b**) 1 h and (**c**) 3 h of annealing; with (1 0 1) orientation after (**d**) 0.5 h, (**e**) 1 h and (**f**) 3 h of annealing; with (1 1 1) orientation after (**g**) 0.5 h, (**h**) 1 h and (**i**) 3 h of annealing.

AFM 3D images of the tungsten surface after annealing at different experimental conditions are shown in Figure 3. We focus on the microstructural evolution of three typical grains with a basic miller index. The morphology and topography of the tungsten grains with (0 0 1), (1 0 1) and (1 1 1) crystal orientations after 0.5 h, 1 h and 3 h of annealing are shown in Figure 3a–c, Figure 3d–f and Figure 3g–i, respectively. From the AFM 3D images, many protrusions appear on the sample surface. The size of the bottom width is obviously larger than that of the height of the protrusions. Although different sizes of the protrusions are formed on the sample surface, the ratio of the radius to the height is larger than 1, *r*/*h* > 1, where *h* and *r* are the bottom radius and height of the protrusions, respectively. So the self-assembly characteristic microstructures are like cones. According to the original AFM data, the height and radius of the self-assembly cones in these grains can be measured and the values are both less than 100 nanometers. From the AFM 3D images, it is obvious that the size of the nanocones increases with increasing the annealing time, which is the opposite for the density of the nanocones. At the same experimental conditions, the density of the nanocones is largest on the grain with the (1 1 1) crystal orientation and smallest on the grain with the (0 0 1) crystal orientation. In fact, when the polycrystalline tungsten is irradiated with energetic ions, the surface morphology always exhibits a dependence on the crystal orientation. Our present results are similar to Garrision’s research on the distinct difference among the grains with three basic miller indexes [27]. In Garrision’s investigation, the surface with the (0 0 1) crystal orientation was found to be most resistant to He^+^ ion erosion. However, other surfaces suffered very serious damage as the crystal orientation deviated away >8° from the (0 0 1) orientation. The grains with (1 0 1) and (1 1 1) crystal orientations had highly serious erosion. Meanwhile, different surface nanostructures were also observed on the surface of the polycrystalline tungsten with different crystal orientations implanted by 30 keV He^+^ ions [28]. In our previous research [29], the grains of the tungsten with various orientations behaved quite differently under a 30 keV focused Ga^+^ ion beam bombardment. The grains with the (0 0 1)direction parallel to the ion beam always maintained a much smoother surface morphology with less mass removal after ion bombardment, indicating a lower sputtering yield.

**Figure 4:**
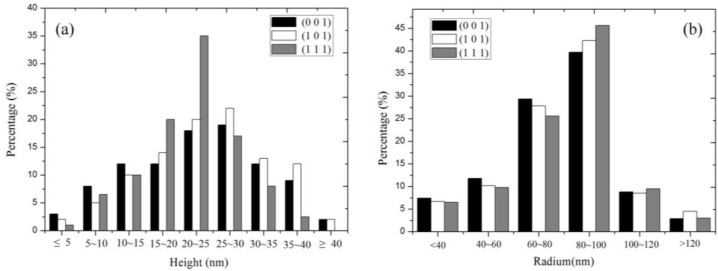
The distribution frequencies of (**a**) height and (**b**) radius of the nanocones formed on the grains with (0 0 1), (1 0 1) and (1 1 1) crystal orientations after post-irradiation annealing for 0.5 h.

The distribution frequencies of the height and radius of the self-assembly nanocones on the tungsten surface after post-irradiation annealing for 0.5 h are shown in Figure 4. In the statistical analysis, more than 200 nanocones at one grain were measured. On the overall distribution trend, for these three typical grains with a basic miller index, the height and radius of the self-assembly nanocones show the characteristics of Gaussian distribution. The height value mainly distributes in the range of 15 to 35 nm, corresponding to the radius value in the range of 60–100 nm. However, in fact, the distribution characteristics are slightly different on the grains with different crystal orientations. For the grain with the (1 1 1) orientation, the distribution frequency of the nanocones with the height in the range of 20–25 nm is about 35%. However, it is only about 18% of the nanocones with that height value on the grain with the (0 0 1) orientation and about 20% on the grain with the (1 0 1) orientation. Further, it cannot be found on the nanocones with a height value over 40 nm on the grain with the (1 1 1) orientation. Statistical results show that the standard deviation of the distribution frequencies of the grain with the (1 1 1) orientation is smallest in these three gains with a basic miller index, which means the dimensions of the self-assembly nanocones on the grain with the (1 1 1) orientation are the most concentrated and uniform. Correspondingly, the standard deviation is largest in the grain with the (0 0 1) orientation. Meanwhile, according to the statistical results, after annealing with the same time, the density of the nanocones is smallest in the grain with the (0 0 1) orientation, largest in the (1 1 1)-oriented grain, and medium in the (1 0 1)-oriented grain.

Apart from the dependence of the grain orientation, the height and the radius of the nanocones also exhibit an obvious dependence on the annealing time. As shown in Figure 3, it can be seen that the size of the self-assembly nanocones on the same grain surface significantly increases with the increasing annealing time. In addition, the annealing causes the combination of the nanocones during their growth. The sharpness of the nanocones decreases with the increasing annealing time as shown in the AFM 3D images, and the effect is quite obvious after 3 h of annealing as shown in Figure 3c,f,i. Moreover, the size and morphology of the nanocones tend to be more uniform with the increasing annealing time.

**Figure 5:**
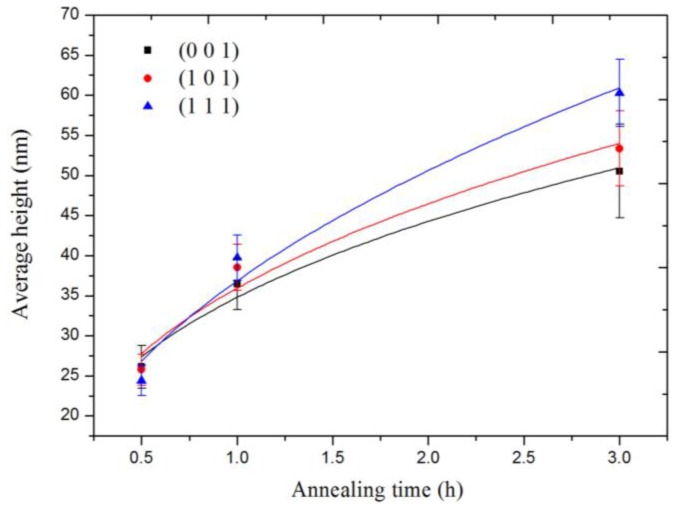
The height *h* of the nanocones on the irradiated tungsten surface with (0 0 1), (1 0 1) and (1 1 1) crystal orientations vs. annealing time.

The quantitatively statistical results of the height *h* of the self-assembly nanocones formed on the grains with (0 0 1), (1 0 1) and (1 1 1) crystal orientations, varying with the annealing time periods, are shown in Figure 5. It indicates that not only the height of the nanocones is obviously different with different crystal orientations, but also the growth rate is different. According to the statistical results, the height value can be fitted quite well by the power-law dependence as a function of the annealing time: for the grain with the (0 0 1) orientation, *h* ∝ *t*^0.347^, for the grain with the (1 0 1) orientation, *h* ∝ *t*^0.370^; and for the grain with the (1 1 1) orientation, *h* ∝ *t*^0.457^. The exponents in the power-law equations are correlated with the crystal orientation. Similar results of the power-law functional relationship for the self-assembly nanostructures induced by ion irradiation have been reported in the published literature [30]. The size of the formed nanodots was proportional to a power-law function which was relative to the ion irradiation parameters including ion flux and fluence. In fact, when an ion beam is used to irradiate materials, the surface roughness is usually measured as a function of time and other system parameters. In addition, the results are used to obtain the exponents of the power-law function that are dependent on these experimental parameters [31,32].

**Figure 6:**
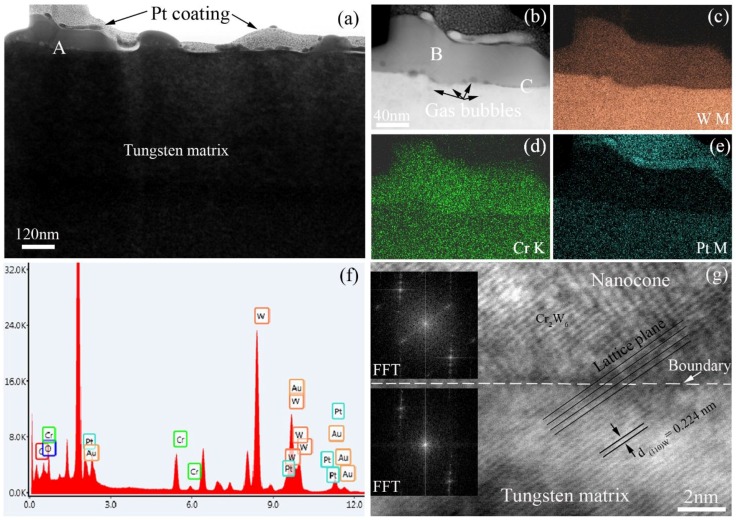
(**a**) Bright field scanning transmission electron microscopy (STEM) image showing the microstructure of the self-assembly nanocones on the irradiated tungsten surface annealed for 3 h; (**b**) High-angle annual dark field STEM image of the nanocone; (**c**–**e**) Element mappings of W M, Cr K and Pt M, respectively; (**f**) Energy dispersive spectrometer (EDS) results of the nanocone; (**g**) High-resolution transmission electron microscopy (TEM) image of the crystal boundary between tungsten matrix and the nanocone.

In order to obtain the detailed microstructure of the self-assembly nanocones formed on the sample surface after He^+^ ion irradiation and then annealing for 3 h, the cross-sectional TEM samples along with the normal direction of the tungsten surface were prepared by in situ FIB. TEM analysis results are shown in Figure 6. The bright field scanning transmission electron microscopy (STEM) image shows the cross-sectional morphology of the nanocones located at the top of the tungsten matrix, as shown in Figure 6a. Some information such as the tungsten matrix, Pt coating and nanocones are labeled in the image. The boundary between the self-assembly nanocones and the tungsten matrix can be obviously observed. Under the STEM model, the color of the nanocones and tungsten matrix is different, which further indicates the chemical composition is different based on the imaging mode of STEM. The chemical composition of the nanocones contains not only tungsten, but also other elements. According to the chemical composition provided by the sale company and further tested by Standard Test Institute of China, the purity of the tungsten is more than 99.8 wt %. The main impurity is chromium, but that is less than 0.2 wt %. Therefore, the element mappings of W, Cr and Pt were tested in the typical nanocone as marked by letter ‘A’ in Figure 6a. The high angle annual dark field STEM image of that nanocone is shown in Figure 6b, which is a high-magnification image. The nanocones should be the combination of two original nanocones. Meanwhile, some gas bubbles with a dark color can be also observed as indicated by dark arrows. The injected helium atoms will migrate, gather, combine and grow to form gas bubbles during the annealing. The gas bubbles distribute not only in the tungsten matrix, but also at the boundary between the nanocones and tungsten matrix.

The results of the element mappings of W, Cr and Pt are shown in Figure 6c–e, respectively. At the location of the nanocone, the color of chromium is shiny, which indicates the nanocone contains chromium atoms. Comparing the content of chromium in the nanocone with that in the tungsten matrix, it can be concluded that the existence of chromium is responsible for the formation of the self-assembly nanostructures on the tungsten surface, and because we did not find the nanocones self-assembled on the sample surfaces of tungsten with 99.999 wt % purity at all of the same experimental parameters including the sample preparation process, irradiation conditions and annealing parameters, except for the natural tungsten. The energy dispersive spectrometer (EDS) results coming from the location of the nanocone marked letter ‘B’ is shown in Figure 6f, which includes the diffraction peaks of W, Cr, Pt, Au, O and C. The diffraction peaks of Au and Pt come from the deposition coating used as a protective layer during the cross-sectional TEM sample preparation. The diffraction peaks of O and C elements are from surface contamination sneaking from the experimental process. Similar EDS results are detected in other nanocones. Figure 6g shows a high-resolution TEM image of the boundary between a self-assembly nanocone and tungsten matrix from the location marked letter ‘C’ in Figure 6b. The FFT images of the nanocone and tungsten matrix are inserted in the left upper side and the bottom left, respectively, which indicates the nanocones grow along with the (1 1 0) crystal plane of tungsten. The nanocones should be the Cr_2_W_6_ compound because the interplanar spacing of the (0 0 4) crystal plane of Cr_2_W_6_ is almost the same as that of the (1 1 0) crystal plane of tungsten, d_(0 0 4)Cr_2_W_6__ = 0.222 nm and d_(1 1 0)W_ = 0.224 nm, which is also proved by the fast Fourier transform (FFT) results of the nanocone and tungsten matrix. The lattice mismatch is less than 0.9%.

When helium ions with low energy are used to bombard the tungsten surface, injected ions penetrate into the sample surface within a finite depth (the maximum depth is approximately 600 nm, as shown in Figure 1) and transfer their kinetic energy by colliding with the substrate atoms, which induces many defects such as vacancies, interstitials, dislocations and voids generated in the irradiated layer. During the post-irradiation annealing, because of the function of the extra energy provided by the high temperature, these irradiation-induced defects may act as nucleation attracting the migration of impure chromium atoms towards the tungsten surface. This gathering process of chromium atoms at the location of surface defects will result in the nucleation of a new compound formation with chromium atoms and tungsten atoms because this process will reduce the system’s energy. Simultaneously, vacancies and interstitials of helium atoms will combine to form complex structures such as He*_x_*V*_y_*, for example He_2_V, He_3_V, He_4_V_2_ (V is short for vacancy) [33,34]. Some studies show that He bubbles are preferentially trapped at the grain boundaries and interfaces during the irradiation and then annealing [22,35,36]. Therefore, the complex structures He*_x_*V*_y_* will form at the small interfaces between the nucleated new compound and tungsten matrix, which increases the defect density near the sample surface. Finally, the migration process of chromium atoms towards surface defects would interact with the trapping process of He*_x_*V*_y_* structures at the surface interface, and the mutual promotion of these two processes induces the formation of the nanocones and then their growth during the further annealing.

The variation of the self-assembly nanocones on the grains with different crystal orientations can be attributed to the mobility difference of the impure chromium atoms and vacancy clusters in the different grains. After helium ion irradiation, the generated defects including interstitials, vacancies and helium-vacancy clusters result from the displacement damage of the atom collision. These defects hop intensively during high-temperature annealing, and may diffuse towards the sample surface or move into the unirradiated part of the tungsten matrix or combine themselves. Tungsten is a body-centered cubic structure, which has three slip families: 12 slip systems in {1 1 0} <1 1 1>, 12 slip systems in {1 1 2} < 1 1 1>, and 24 slip systems in {1 2 3} <1 1 1>. They all have the same slip direction of <1 1 1>. This is consistent with a previous investigation by Xu et al. [37]. In their work, tungsten was exposed to D plasma. Blisters occurred most strongly for the grains with the normal direction <1 1 1>, which is the preferred slip direction of bcc metals. They demonstrated that D-defect complexes near the surface in (1 1 1)-oriented grains grew most easily by plastic deformation to form blisters. Moreover, according to the results of the literature report [38], the recoil energy of the vacancy migration in the <1 1 1> direction is minimal, so the defect clusters created by ion irradiation and the chromium impurity transfer along with the <1 1 1> direction most easily. Therefore, these two factors lead to the phenomenon that most nanocones self-assembled on the grain surface with the (1 1 1) orientation. Therefore, the mechanism of the self-assembly nanocones is due to a complex combined effect.

## 4. Conclusions

The polished polycrystalline tungsten (purity, >99.8%) with 3 nm surface roughness was first irradiated by He^+^ ions with a series of energies degraded from 200 keV to 20 keV and corresponding ion fluence at room temperature, and then annealed at 900 °C for different lengths of time. The morphology and microstructure were characterized using AFM, SEM and TEM. The cross-sectional TEM samples were prepared using in situ FIB. The experimental results showed that the self-assembly nanocone structures were induced by the combined effect of He^+^ ion irradiation, the annealing process and the chromium impurity. The distribution characteristics, density and morphology of the self-assembly nanocones exhibit a distinct difference related to the crystal orientations that played an important role in the formation of the nanocones. The highest density of the nanocones was observed on the grain surface with the (1 1 1) orientation, with the smallest density for that with the (0 0 1) orientation, and a medium value for the (1 0 1)-oriented grain. The size of the nanocones increased with the increasing annealing time which met a power-law relationship. He^+^ ion irradiation and the post-annealing process caused the migration of impure chromium atoms. Chromium migration was thought to result from near-surface He trapping at the intrinsic defect sites. Under the action of temperature, the protrusions finally evolved into nanocones. In particular, the excellent physical and chemical stability of tungsten allowed tungsten-based devices at nanoscale to be made for high-temperature and high-power operation conditions.
